# 胸膜肺母细胞瘤临床病理分析

**DOI:** 10.3779/j.issn.1009-3419.2010.05.31

**Published:** 2010-05-20

**Authors:** 仕高 陈, 世凤 王, 俊 高, 尚福 张

**Affiliations:** 1 610041 成都，四川大学华西医院病理科 Department of Pathology, West China Hospital of Sichuan University, Chengdu 610041, China; 2 610081 成都，成都铁路分局医院病理科 Department of Pathology, Chengdu Railway Branch Hospital, Chengdu 610081, China

**Keywords:** 胸膜肺母细胞瘤, 免疫组化, 病理诊断, 鉴别诊断, Pleuropulmonary blastoma, Immunohistochemistry, Pathologic diagnosis, Differential diagnosis

## Abstract

**背景与目的:**

胸膜肺母细胞瘤是一种罕见的且有其独特临床病理特征的恶性肿瘤，本文旨在探讨胸膜肺母细胞瘤的临床病理学特点及鉴别诊断等。

**方法:**

收集5例胸膜肺母细胞瘤，复习患者临床资料，进行组织学及免疫组化观察，并结合文献复习分析。

**结果:**

5例胸膜肺母细胞瘤患者年龄为21个月-47个月，平均为32.8个月；肿瘤主要位于胸腔，4例伴有胸腔积液。组织学观察：Ⅰ型胸膜肺母细胞瘤1例，呈单纯囊性；Ⅱ型胸膜肺母细胞瘤2例，呈囊实性，实性区伴横纹肌分化及灶性软骨样结节；Ⅲ型胸膜肺母细胞瘤2例，完全呈实性，可见间变性未分化肉瘤样成分。免疫组化染色显示肿瘤细胞呈Vimentin（+），部分肿瘤细胞呈Desmin和Myogenin（+），软骨样结节呈S-100（+）；PCK、EMA、CD99等均（-）。

**结论:**

胸膜肺母细胞瘤是一种罕见的主要发生于婴幼儿肺和胸膜的高侵袭性恶性肿瘤，各亚型均有其临床病理学特点。诊断方面应与肺先天性囊性腺瘤样畸形、胚胎性横纹肌肉瘤等良恶性病变鉴别。

## Abstract (en)

**Background and objective:**

Pleuropulmonary blastoma (PPB) is a rare malignant tumor with unique clinicopathological features. The aim of this study is to investigate the clinicopathological features, the diagnosis and differential diagnosis of pleuropulmonary blastoma.

**Methods:**

Five cases of PPB were analyzed by light microscopy, immunohistochemistry and their clinical data, and the relative literatures were reviewed.

**Results:**

Five cases of patients suffered from PPB were aged from 21 to 47 months (mean 32.8 months). Most of the masses were located in the thoracic cavities and 4 cases accompanied with pleural effusions. Histologically, these tumors included 1 case of type Ⅰ PPB which showed pure cystic architecture; 2 cases were type Ⅱ PPB which showed cystic and solid masses accompanied with rhabdomyoblastic differentiation and nodules of cartilage; the other 2 cases were type Ⅲ PPB and characterized by absolute solid masses with anaplastic undifferentiated sarcomatous components. Immunohistochemical studies showed that tumor cells were positive for Vimentin and some for Desmin and Myogenin, the nodules of cartilage were positive for S-100. The tumor cells were negative for PCK, EMA and CD99.

**Conclusion:**

Pleuropulmonary blastoma is a rare and highly aggressive malignancy arising in the lung and pleural of infancy and early childhood. The type Ⅰ, Ⅱ and Ⅲ PPB have unique clinicopathological features respectively. This kind of tumor should be distinguished from some benign and malignant diseases such as congenital cystic adenomatoid malformation (CCAM) and embryonal rhabdomyosarcoma.

胸膜肺母细胞瘤（pleuropulmonary blastoma, PPB）是一种罕见的主要发生于婴幼儿肺和胸膜的恶性肿瘤，又称为儿童型肺母细胞瘤，分为Ⅰ型、Ⅱ型和Ⅲ型，其临床和病理上均有其独特表现。现将收集的5例PPB并结合文献对其临床及病理学特点等进行分析。

## 1 材料与方法

### 1.1 材料

本组5例PPB中，4例为四川大学华西医院1999年-2009年间外检病例，1例为会诊病例（表 1）。

**1:** 5例胸膜肺母细胞瘤的临床及病理特征 Clinicopathological features of 5 cases of pleuropulmonary blastoma

No.	Age (month)/sex	Clinical symptom	Sites and sizes	Macroscopy	Pathologytypes	Follow-up
1	30/F	Cough, expectoration, dyspnea	Inferior lobe of left lung (4 cm × 3 cm)	Cystic mass	Ⅰ	NEDfor5 monthsafter surgery
2	21/F	Ecphysesis	Filled the left thoracic cavity	Multicysticmass	Ⅱ	DOD at 4 months after surgery
3	24/F	Dyspnoea, emesis	Left thoracic cavity (1.5 cm × 1 cm × 1 cm)	Solid, fish-like	Ⅱ	DODatl month after surgery
4	47/F	Cough, breath lessness, low-grade fever	Left thoracic cavity (11 cm ×9 cm × 9 cm)	Solid, fish-like, accompanied byhaemorrhage and neaosis	Ⅲ	Lost
5	42/F	Cough, fever	Filled the right thoracic cavity	Solid, fish-like, accompanied by necrosis	Ⅲ	Lost
F: female; NED: no evidence of disease; DOD: dead of disease.						

### 1.2 方法

5例PPB标本均用4%甲醛固定，石蜡包埋，行HE染色，部分病例选用Vimentin、Desmin、Myogenin、Myoactin、S-100、PCK、EMA和CD99等行免疫组化染色，免疫组化采用SP法，操作按试剂说明书进行，抗体及试剂均购自福州迈新生物技术开发有限公司。

## 2 结果和分析

### 2.1 临床资料

5例PPB临床及病理特征见表 1。5例均为女性患儿，年龄为21个月-47个月，平均年龄32.8个月，均无相关疾病家族史。以咳嗽、咳痰、气急等呼吸系统症状为主要临床表现，有的伴有发热、呕吐。影像学方面，CT检查提示4例伴有胸腔积液，3例伴有纵隔移位，2例提示有肺不张；其中第1、2例病变区为高透亮气体影，似肺大泡；第4、5例示胸腔内巨大软组织密度肿块影，边界不清，其内密度不均等。术中见4例肿瘤位于胸腔，1例肿瘤位于左肺下叶外侧近胸膜处，其中第2、4例肿瘤组织与胸壁粘连，第5例肿瘤组织与胸壁、肝脏、心脏及右肺粘连。

### 2.2 病理检查

#### 2.2.1 肉眼观察

5例PPB均为单发，肿瘤小者1.5 cm×1 cm×1 cm，大者占满整个胸腔。其中1例为单房囊性包块，1例为多房囊性包块，3例为实性包块。实性包块切面均为灰白色、鱼肉状，伴有出血及坏死。

#### 2.2.2 光镜观察

5例中，第1例为Ⅰ型PPB，肿瘤组织呈囊性（图 1A），与正常肺组织界限清楚，囊壁内衬成熟的呼吸道上皮细胞；沿上皮下见原始间叶样小细胞聚集，细胞圆形或短梭形，似葡萄状肉瘤生发层（图 1B）。第2、3例为Ⅱ型PPB，其中第2例肿瘤组织呈囊实性，囊性区呈典型Ⅰ型PPB组织像，实性区大部向横纹肌分化，部分区域肿瘤细胞异型明显，另见散在幼稚的圆形和梭形细胞，并见陷入的良性上皮成分（图 1C）；第3例主要由实性肿瘤组织构成，肿瘤细胞为小圆形或短梭形原始间叶样细胞，可见灶性软骨样结节（图 1D），并见出血、坏死及血栓形成，另见少许囊壁成分，其组织像同Ⅰ型PPB。第4、5例为Ⅲ型PPB，完全由实性肿瘤组织构成，均可见原始间叶样细胞；两例均伴横纹肌肉瘤分化和灶性软骨样结节，其中第5例见大片坏死，部分区域呈间变性未分化肉瘤样组织像（图 1E）。

**1:**
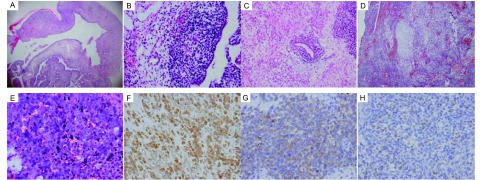
PPB的病理组织学图像。A：Ⅰ型PPB，肿瘤组织呈囊性（HE, ×40）；B：上皮下似葡萄状肉瘤生发层的原始间叶细胞（HE, ×100）；C：示陷入的良性上皮成分（HE, ×100）；D：示软骨样结节分化（HE, ×40）；E：示间变性未分化肉瘤组织像（HE, ×400）；F：肿瘤细胞示Vimentin阳性（SP, ×200）；G：部分肿瘤细胞示Desmin阳性（SP, ×200）；H：部分肿瘤细胞示Myogenin阳性（SP, ×100） Pathohistologic pattern of PPB. A: Type Ⅰ PPB appears as cystic structure (HE, ×40); B: Primitive mesenchymal cells like cambium layer of botryoid sarcoma dispersed beneath the epithelium (HE, ×100); C: Benign epithelium entrapped within the tumor (HE, ×100); D: Nodules of cartilage in the tumor (HE, ×40); E: Histologic pattern like anaplastic undifferentiated sarcoma (HE, ×400); F: Tumor cells positive for Vimentin (SP, ×200); G: Partial tumor cells positive for Desmin (SP, ×200); H: Partial tumor cells positive for Myogenin (SP, ×100)

### 2.3 免疫表型检测

5例肿瘤细胞呈Vimentin（+）（图 1F），其中第2、4、5例肿瘤细胞向横纹肌分化者呈Desmin（+）（图 1G）和Myogenin（+）（图 1H），第4例部分肿瘤细胞Myoactin（+）。第3、4、5例中软骨样结节细胞呈S-100（+）。5例肿瘤细胞均对CD99、PCK、EMA等呈阴性表达。

## 3 讨论

PPB又称为儿童型肺母细胞瘤，是一种罕见的主要发生于婴幼儿肺和胸膜的恶性肿瘤。1989年PPB被确定为一种独立的病理实体，WHO（2003）肺组织肿瘤分类将其归入肺间叶源性肿瘤，迄今为止大约有300例病例报告^[1]^。目前将PPB分为3型：Ⅰ型（多囊型）、Ⅱ型（多囊伴实性结节型）和Ⅲ型（实体型）。与经典的肺母细胞瘤不同，PPB主要发生于婴幼儿，约93%的患者小于6岁^[2, 3]^，发生于成人者目前仅有4例报道^[4]^，性别上无明显差异。Ⅰ型PPB平均发病年龄为10个月，Ⅱ型为36个月，Ⅲ型为44个月^[5]^。Ⅰ型PPB多局限于肺内和/或脏层胸膜，Ⅱ型和Ⅲ型则多累及肺外如壁层胸膜、纵隔和膈肌等。PPB患者主要表现为咳嗽、呼吸困难、发热、胸部或腹部疼痛等症状，另有报道^[6, 7]^可出现张力性气胸及脓胸。影像学方面，CT检查常表现为囊实性混杂密度占位，囊内可含多少不等气体，也有类圆形或形态不规则软组织密度占位，其内可见不规则略低密度区；增强扫描实性部分有不同程度的增强，低密度区不强化；常合并胸腔积液、肺不张、肋骨破坏等。

肉眼观察发现，Ⅰ型PPB呈多房囊性结构；Ⅱ型呈囊实性，囊壁较厚，可见突入囊腔的实性结节；Ⅲ型病例为完全实性的包块，切面质软、鱼肉样，常见出血和坏死。本组肿瘤大体表现与此一致，其中第1、2例手术中囊腔内有气体溢出，文献中未见报道，提示肿瘤可能与气道相通。组织学观察发现，Ⅰ型表现为囊壁内衬以成熟的呼吸道上皮细胞，上皮下见原始间叶细胞，肿瘤细胞小而圆，可有横纹肌母细胞分化；Ⅱ型表现为在Ⅰ型基础上出现了灶性的实性区；Ⅲ型完全由实性区构成。Ⅱ、Ⅲ型PPB实性区由幼稚的圆形或梭形细胞构成，可以伴有分化的和/或间变的肉瘤样成分，包括胚胎性横纹肌肉瘤（embryonal rhabdomyosarcoma, ERMS）、纤维肉瘤、间变性未分化肉瘤和以上肿瘤的混合成分，并可出现幼稚的软骨样结节，还可伴有脂肪肉瘤分化^[8]^。在肿瘤的早期，正常发育中的肺组织与肿瘤之间存在过渡，表现为肺泡间隔中的间叶性细胞增殖导致其均匀扩张增厚^[3]^，此即形成Ⅰ型PPB的组织学变化。随着肿瘤的进展，增殖的间叶性细胞形成实性结节突入囊腔，大体上即表现为囊实性结构，此即Ⅱ型PPB；最后，实性结节完全取代囊性区，至此进展为Ⅲ型PPB。在此演进过程中，增殖的间叶细胞可出现横纹肌母细胞、软骨结节等分化，且细胞异型性逐渐明显。免疫表型检测显示肿瘤细胞呈Vimentin（+）表达，随着原始间叶细胞出现分化则有相应的免疫表型表达如Desmin、Myogenin、MSA、SMA等。CK、EMA（+）表达仅见于肿瘤组织中陷入的非肿瘤性呼吸性上皮细胞。

PPB的诊断和鉴别诊断主要依靠病理形态表现和临床资料如患者年龄等。PPB多见于6岁以下儿童，胸部影像学检查可见肺周边和/或胸膜孤立性占位，可以是囊性、实性或囊实性。对于Ⅰ型PPB，由于其与某些肺先天性囊性病变在影像学、大体检查方面均呈单纯囊性结构，因此只有病理组织检查才可区分。而肺先天性囊性腺瘤样畸形（congenital cystic adenomatoid malformation, CCAM）和Ⅰ型PPB在组织学改变上可有重叠^[3, 9]^，且Ⅰ型PPB囊壁被覆上皮下诊断性的原始间叶细胞分布常比较局限，导致它们之间区别极为困难。但Ⅰ型PPB或多或少有原始间叶细胞分化，甚至有软骨、纤维母细胞样、脂肪样分化，肿瘤细胞常有核型异常，其细胞免疫表型检测显示p53较强阳性表达，而CCAM与之不同，可资鉴别^[10]^。Ⅱ型PPB肿瘤组织实性区及Ⅲ型PPB需与以下肿瘤鉴别：①ERMS：PPB常有横纹肌母细胞分化，甚至以ERMS成分为主，容易将其诊断为ERMS。但ERMS为单向性肌源性分化，无原始间叶细胞及软骨样成分^[1, 11]^，且多发生在鼻腔、泌尿道、阴道等有腔器官，很少发生于胸膜，肿瘤组织内无其它肉瘤和上皮成分。②某些恶性小圆细胞肿瘤：当PPB以原始间叶细胞成分为主时，需与原始神经外胚叶肿瘤/Ewing’s肉瘤、Askin瘤及促纤维增殖性小圆细胞肿瘤、转移性肾母细胞瘤、淋巴瘤等鉴别。此时，临床资料、免疫表型检测甚至电镜、遗传学检查有帮助。③经典型肺母细胞瘤：当PPB中含有陷入的上皮成分时，可与经典型肺母细胞瘤混淆。但经典型肺母细胞瘤的上皮成分为类似于胎儿性腺癌的恶性原始上皮成分，而PPB的上皮成分为少量的正常的呼吸性上皮。④滑膜肉瘤：滑膜肉瘤常发生于青年人四肢大关节旁的深部软组织，与PPB不同，瘤组织中始终可找到双向分化的特征，且上皮成分亦为肿瘤性成分，一般不见原始间叶细胞及横纹肌母细胞分化，肿瘤细胞异型性小且可弥漫性表达bcl-2蛋白。⑤肉瘤样间皮瘤：肉瘤样间皮瘤发病年龄较大，一般无PPB常见的原始间叶细胞及横纹肌母细胞样成分，肿瘤细胞免疫组化染色钙结合蛋白抗体（calretinin）和间皮细胞抗体（mesothelial cell, MC）阳性支持间皮瘤的诊断。⑥其它恶性肿瘤：当PPB含有多种肉瘤分化甚至间变的肉瘤样成分时，需与平滑肌肉瘤、恶性间叶瘤、未分化肉瘤、恶性畸胎瘤、黑色素瘤及其它胸膜发生的肉瘤鉴别。

PPB的生物学行为与肿瘤类型有关，Ⅰ型PPB极少发生转移，而25%以上的Ⅱ、Ⅲ型PPB可发生转移^[12, 13]^，主要转移至中枢神经系统^[11]^和骨，且Ⅲ型转移率高于Ⅱ型^[5]^。据文献^[3, 14]^报道，Ⅰ型PPB的总生存率为85%-90%，而Ⅱ、Ⅲ型PPB总生存率分别约为60%和45%。PPB的治疗方法包括手术切除、化疗和放疗等。对于肺的囊性病变，由于术前不能排除Ⅰ型PPB的可能性，应一律外科手术切除^[15]^。对于Ⅰ型PPB，若无肺外累及则行单纯肿瘤手术切除^[16]^。也有报道^[14, 17]^认为术后化疗可降低Ⅰ型PPB复发的风险并提高生存率。对于Ⅱ、Ⅲ型PPB应尽量完整切除肿瘤，并辅以化疗。对于不能手术切除者可先行化疗以缩小肿瘤体积使其可被切除^[8]^。术后有残留病灶者可加放疗，对于复发病例可考虑在大剂量化疗后行自体干细胞移植^[1, 17]^。但对于幼儿是否附加放疗存在争议，因为其可导致严重的并发症^[8]^。对于PPB患者术后应使用胸部CT检查严格随访3年以上^[16]^。
